# Acute Effects of Supervised Making Weight on Health Markers, Hormones and Body Composition in Muay Thai Fighters

**DOI:** 10.3390/sports8100137

**Published:** 2020-10-16

**Authors:** Roberto Cannataro, Erika Cione, Luca Gallelli, Natale Marzullo, Diego A. Bonilla

**Affiliations:** 1 Department of Pharmacy, Health and Nutritional Sciences, University of Calabria, 87036 Rende, Italy; erika.cione@unical.it; 2 Clinical Pharmacology and Pharmacovigilance Operative Unit, Department of Health Science, University of Magna Graecia, Mater Domini Hospital Catanzaro, 88100 Catanzaro, Italy; gallelli@unicz.it; 3 Italian Boxing Federation, 06088 Assisi, Italy; natalemarzullonflab@gmail.com; 4 Research Division, DBSS International SAS, Bogotá 110861, Colombia; dabonilla@dbss.pro; 5 kDNA Genomics^®^, University of the Basque Country UPV/EHU, 20018 San Sebastián, Spain; 6 Research Group in Biochemistry and Molecular Biology, Universidad Distrital Francisco José de Caldas, Bogotá 110311, Colombia; 7 Research Group in Physical Activity, Sports and Health Sciences (GICAFS), Universidad de Córdoba, Montería 230002, Colombia

**Keywords:** weight reduction diet, glycogen depletion, dehydration, blood chemical analysis, body composition, combat sports, rapid weight loss

## Abstract

Making weight is a practice often used in combat sports. This consists of a rapid weight loss (RWL) and a subsequent rapid weight gain (RWG) in the days preceding competition. However, this practice is often carried out based on anecdotal information provided by ex-athletes or non-professionals, which has led to several adverse events. This study aimed to assess the acute effects of a supervised nutritional period of RWL/RWG on health markers, hormone concentrations, and body composition. We performed a single-arm repeated-measures (baseline, after RWL and after RWG) clinical trial with twenty-one (8F:16M) Italian Muay Thai fighters. Body mass was significantly lower after the RWL (−4.1%) while there was a significantly higher glucose availability after RWL and RWG. Blood urea nitrogen, lipid profile, and creatinine were within the normal range after RWL/RWG. Testosterone decrease significantly after RWL and RWG in the men group. Male fighters had a significant reduction in thyroid-stimulating hormone concentration after the RWL and RWG intervention, but no change was found in women at pre-competition. Bioelectrical parameters were almost fully restored after RWG. An evidence-based and individualized nutrition methodology reduces the adverse events after an RWL and RWG practice, although the impact on the hormonal profile is inevitable.

## 1. Introduction

Several sports classify the athletes by weight classes; mainly combat sports (such as Muay Tai and Boxing) [1]. However, jockeys, power lifters, bodybuilders, gymnasts, and fitness practitioners are among the individuals that are subjected to certain body mass criteria or weight classification [2]. In this sense, the colloquially called “making weight” (or “weight-cutting”) is a common practice that is not exclusive for Olympic sports (such as boxing, wrestling, or taekwondo) but also used in sports disciplines like kickboxing, MMA, and jujitsu [3,4]. This practice is also frequently reported in powerlifting and bodybuilding [5,6]. Making weight is characterized by a rapid weight loss (RWL) period and a subsequent rapid weight gain (RWG) in the days before competition [7]. These practices differ across each sport, depending on the distance between the weighting procedure and the match, so the RWL could start 2–3 days before the competition and the RWG could last from 1 to 18 h [8]. It has been reported that combat sports athletes have performed this practice at least once in life [9,10], but this methodology is also known among amateur fighters and it is applied from two to eight times per year (personal communication and experience within the Italian Muay Thai and Italian Boxing federations). The goal of cutting weight is to be able to compete in a lower-weight category than one of the regular season, which would translate into a physical advantage; notwithstanding, scientific literature has demonstrated a reduction in sports and psychological performance after drastic RWL and RWG [8,11]. The issues of the making weight practice encompass not only the frequency over the year but also that is operated based on anecdotes and non-professional supervision, which have called the attention of health professionals due to the alarming potential adverse events [12]. Abuse of pharmacological drugs (e.g., diuretics), large restrictions in energy intake, less muscle recovery, and extreme dehydration methods are strategies frequently used by fighters [13,14]. In fact, the common belief of the mandatory making weight practice within the “ring/cage community” results in a self-administered procedure, which increases notably the risk of relative energy deficiency, severe dehydration, and potential acute kidney injury and health risk [15,16]. There have been several episodes of serious side effects and even death in athletes that aimed to make weight before fight events [1]. Considering the harmful effects of RWL outlined in the existing literature, it is important to determine and monitor an athlete’s minimal competitive body mass to prioritize the health and safety of the athlete [11].

Thus, taking into account the potential benefit of competing with higher body mass and due to the reported side effects of the RWL and RWG in several combat athletes, it is necessary to optimize the methodology of making weight according to exercise physiology and sports nutrition principles (i.e., controlled energy restriction, optimal protein intake, better food sources, and use of nutritional supplements) [17]. In this regard, a recent systematic review by Matthews et al. [8] concluded that there is insufficient evidence to substantiate the use of RWG as a proxy for RWL, hence more studies under standardized conditions and professional supervision are needed in order to provide solid evidence that might improve this practice and avoid the above-mentioned health risks.

To our knowledge, there is no data about the acute effects of a nutritional intervention to “making weight” under professional supervision on health markers, hormones and body composition in Italian elite Muay Thai fighters. Therefore, the aim of this study was to evaluate if a short-term controlled RWL and RWG might be operated in a more effective and safe manner. We hypothesized that our supervised nutritional intervention based on less dangerous strategies (i.e., individualized energy intake based on maintenance calories, controlled macronutrient distribution, quality, and nutrient timing) and the use of certain nutritional supplements (i.e., anti-inflammatory and pro-resolution lipid mediators, and electrolyte complex) would have no side effects on health and body composition but plausible fluctuations on hormone changes might occur.

## 2. Methods

### 2.1. Trial Design

**Figure 1:**
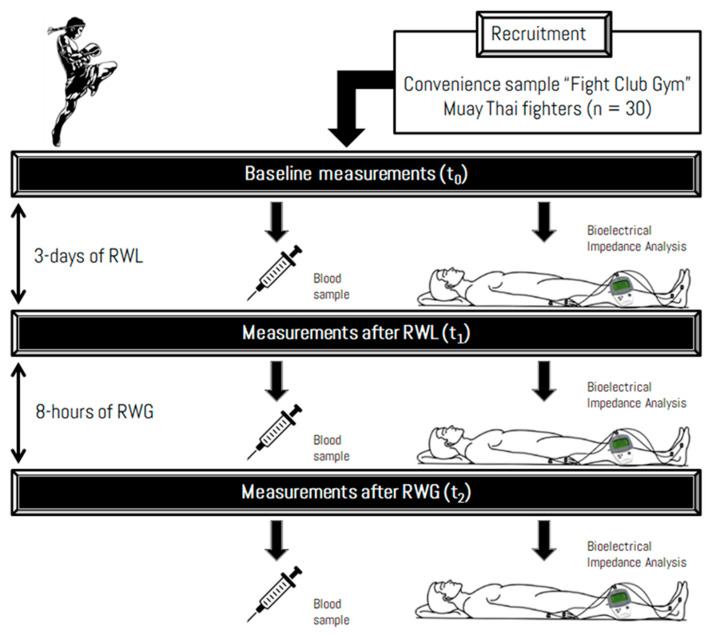
Schematic representation of the study design.

This was a single-arm with a repeated-measures clinical trial to assess the acute effects of supervised RWL and RWG on health markers, hormone concentrations, and body composition in high-level Italian Muay Thai fighters. All variables were measured at baseline (t_0_), after the RWL (t_1_) and after the RWG (t_2_) (Figure 1).

#### 2.1.1. Participants

High-level Muay Thai athletes attending the Fight Club ASD (Rende (CS), Italy) enrolled in this study. The inclusion criteria were as follows: (i) fighters between the ages of 18 and 40-years-old; (ii) that have completed at least three official fights; (iii) having done the “making weight” strategy at least once; (iv) apparently healthy. Any pathology was considered as an exclusion criterion (including but not limited to diabetes, hypertension, renal diseases, liver dysfunction, and neoplastic diseases in the 5-year period prior to the study), besides a medical record of alcohol or drug abuse. The fighters declared having done the making weight autonomously, with no organization and in an unsupervised manner. All participants were informed about the experimental protocol and the potential associated risks since, even if short, the nutritional intervention was very restrictive. An experimental written informed consent was obtained from participants according to the standards of ethical practice as outlined in the declaration of Helsinki [18]. The ethical approval of this research was granted in accordance with the Regional Ethics Committee of the University of Magna Graecia (#120-18052018, Catanzaro, Italy). The selection period was performed from January 2018 to March 2018, while the experimental procedure took place in June 2018.

#### 2.1.2. Intervention Procedures

Before the initiation of this study the participants were contacted by the researchers to receive general instructions of both the process to track daily food intake and the experimental procedure. The previous week of the intervention was implemented to record all diet intakes in order to determine maintenance calories. During the first visit to the laboratory, a blood sample was taken and the baseline measures of all variables were performed. After this, female and male fighters started a supervised three-day nutritional intervention period of RWL and, subsequently, 8 h of RWG, based on the time course of muscle glycogen supercompensation [8,19] and the time lasting from weight to match, respectively. The other two visits to the laboratory took place after RWL and RWG. All measures were performed approximately between 08:00 and 13:00 at 25 °C.

##### Anthropometry

All anthropometric data were collected during the first visit to the laboratory. Body mass was measured using a medical digital scale to nearest 100 g (Seca 878, Hamburg, Germany). To measure the stature, a fixed adult stadiometer was used (Wunder, Trezzo sull’Adda/MI, Italy).

##### Nutrition Intervention

Each subject received a personalized nutrition program based on the maintenance calories. The supervised RWL intervention was set as −1000 kcal × day^−1^ of daily intake for 3 days. The macronutrient distribution was as follows: CHO < 30 g × day^−1^, PRO 2 g × kg BM × day^−1^, and LIP 0.5 g × kg BM × day^−1^. All participants were supplemented with 3 g × day^−1^ of Omega 3 fatty acids (1.8 g of DHA and 1.2 g of EPA), 1 g × day^−1^ of vitamin C (as ascorbic acid in two separate doses to take in the morning and in the evening), a polyphenol complex that supplied 400 mg × day^−1^ of active ingredients (100 mg of olive leaf extract, 100 mg of Curcuma longa powder, 50 mg of astaxanthin, and 150 mg of red berries extract), and a complex of organic salts once a day (200 mg of potassium carbonate and 200 mg of potassium citrate). The goal of this strategy was to deplete muscle and liver glycogen concentration, which will, in turn, reduce fluid content in a 1:3 ratio (e.g., if 500 g of glycogen are reduced, it would have decreased between 1500 and 2500 mL of fluid content), which will result in a total reduction in body mass of approximately 2–3 kg, as it has been reported previously [8,19]. Ideally, this would be recovered after the subsequent overfeeding, allowing an optimal performance. In this sense, the 8 h of the RWG protocol encompassed the following average distribution: CHO 4.5 g × kg BM × day^−1^, PRO 1.75 g × kg BM × day^−1^ and LIP 0.65 g × kg BM × day^−1^. Every 2 h, participants were asked to consume high digestible (high GI) meals that were dense in calories, which were prepared and provided to the athletes. The preferred choices were rice, pasta, and white bread. The athletes were also asked to drink at least 250 mL of calorie-free flavored water every 45 min, and keep the same nutritional supplementation. The athletes were not forced to drink or eat; in case they could not do it they were invited to do so later, but compliance was high. All dietary supplements were provided by LightFlow Technology S.r.l. (L’Aquila, Italy).

##### Exercise Regimen

The fighters normally trained 2–4 h per day before the intervention; however, throughout this study, all athletes performed < 2 h of low-intensity cardiovascular training (approximately 60% of VO_2max_) in the RWL phase.

### 2.2. Outcomes

#### 2.2.1. Primary Outcome Measures

The considered primary outcomes were health markers and hormones concentrations. A total of 10 milliliters (10 mL) of a blood sample from the forearm veins were collected in 15 mL tubes containing ethylenediaminetetraacetic acid (EDTA, 10 mM) anticoagulant and were stored at 4 °C by a certified researcher at the University of Magna Graecia. After overnight fasting, peripheral blood samples were collected in the morning (30 min post-awakening) at the baseline, after RWL, and after RWG. Glucose (mg/dL), total cholesterol (mg/dL), high-density lipoprotein cholesterol (HDL-c, mg/dL), low-density lipoprotein cholesterol (LDL-c, mg/dL), triacylglycerol (TG, mg/dL), blood urea nitrogen (BUN, mg/dL), and creatinine (mg/dL-1) concentrations were quantified by standardized protocols with the ADVIA 1800 Chemistry System (Siemens Healthcare GmbH, Erlangen, Germany). Plasma testosterone (pg/mL) and thyroid-stimulating hormone (TSH, mIU/mL) concentrations were quantified by direct chemiluminescence using acridinium ester technology with the ADVIA Centaur XP Immunoassay System according to manufacturer’s protocols (Siemens Healthcare GmbH, Erlangen, Germany).

#### 2.2.2. Secondary Outcome Measures

Body composition was measured by bioelectrical impedance analysis (BIA) using a single-frequency (50 kHz) tetrapolar electrical ImpediMED DF50 device (ImpediMed Limited, Pinkenba, Australia) [20]. Participants were required to remain in the supine position for 10 min, while a constant current was applied via adhesive electrodes placed on the wrist and hand of one arm, and on the ankle of the foot on the same side of the body. The data were interpolated by the algorithm model of the device, which displayed active tissue mass as body cell mass (%), total body water (TBW, %), intracellular water (ICW, %), extracellular water (ECW, %), and whole-body phase angle (°). The phase angle is defined as the arctangent of the reactance to resistance ratio and is a considered index of cellular integrity by describing the angular shift between voltage and current sinusoidal waveforms [21]. A protocol for the measurement of body composition by BIA was standardized according to manufacturer’s recommendations: (i) patient was informed prior to the visit to come normally hydrated; (ii) voided (empty bladder); (iii) with no exercise performed 2 h prior to the measurement; (iv) no caffeine ingestion 2 h prior to reading; (v) no alcohol 12 h prior to reading.

#### 2.2.3. Sample Size

After the announcement to participate in this study, there were 30 potential fighters for eligibility from the available population of the Fight Club Gym; however, one did not meet inclusion criteria and five declined to participate. Due to the low sample size, and to compare sex-related aspects, we did not include a control group and based our analysis on within-subject effects.

### 2.3. Statistical Methods

The descriptive statistics are expressed as mean ($\bar{x}$) and standard deviation (SD) with the 95% CI. To determine statistical significance, we examined the 95% CIs for the difference between the mean change scores (Δ_1_ = t_1_ − t_0_, Δ_2_ = t_2_ − t_1_, and Δ_3_ = t_2_ − t_0_). The 95% CI was bias-corrected and accelerated with BCa correction to the resampling bootstrap distributions of the effect size (ES). If the 95% CI excludes zero, the difference will attain significance at the *p* < 0.05 level. ES was calculated as unbiased Cohen’s d (d_unb_). Percentages of change were calculated according to the formula: (post-pre)/pre) × 100. Multi-paired Cumming estimation plots were generated to display the repeated measures data in a factorial design, where two groups (female and male) were measured across three-time points (baseline [t_0_], after RWL [t_1_] and after RWG [t_2_]). A difference-in-differences (Diff-in-Diff) analysis was performed to compare changes in the outcome variables across the three-time points between the female and male. The statistical analyses were performed with the IBM SPSS version 26 (IBM Corp., Armonk, NY, USA) and the Exploratory Software for Confidence Intervals ESCI [22]. Estimation graphics were generated with the developed DABEST ‘Data Analysis using Bootstrap-Coupled ESTimation’ v0.3.0 software library [23] within the R statistical computing environment v4.0.2 [24].

## 3. Results

**Figure 2:**
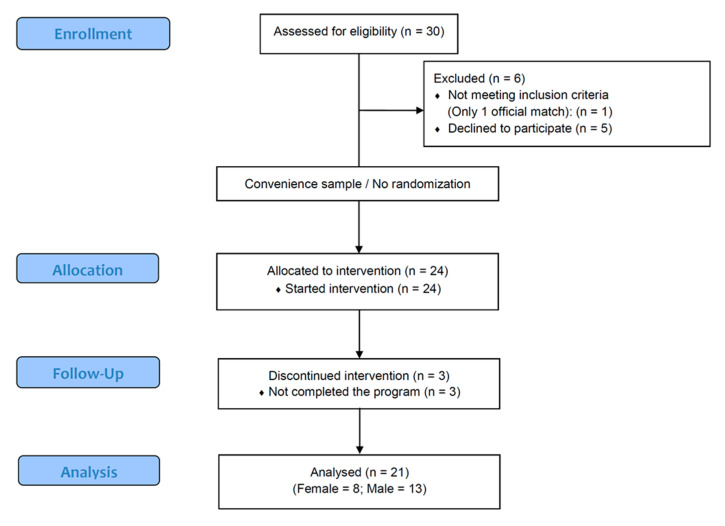
CONSORT flow diagram.

In total, 21 (8F and 16M) high-level Italian Muay Thai fighters (25.80 ± 2.52 years; 68.03 ± 11.56 kg; 1.71 ± 0.08 m; 22.29 ± 2.17 kg·m^−2^) completed this study. All initial female athletes completed the study, while three men did not complete the nutritional intervention. Figure 2 shows a flow chart (CONSORT).

### 3.1. Baseline Data

Table 1 shows the characteristics of the participants by sex at baseline. The participants that were included in this studied had 4.35 ± 1.69 years of training experience. In average, they had participated in 6.9 ± 3.18 sanctioned MMA bouts, with 3.6 ± 2.09 victories and 3.3 ± 1.49 defeats.

#### 3.1.1. Outcomes

The results of all variables are expressed as Δ (SD) [95% CI]; d_unb_ [95% CI] and presented in Table 2.

#### 3.1.2. Rapid Weight Loss (Δ_1_)

As expected, body mass was significantly lower than baseline in all participants after the RWL (Δ_1_ = −2.7 (0.9) [−3.1, −2.3] kg; d_unb_ = −0.237 [−0.329, −0.163]), with an average percentage of change of −4.1%. There was a body mass reduction of 3.5% in female (Δ_1_ = −2.0 (0.4) [−2.3, −1.6] kg; d_unb_ = −0.309 [−0.533, −0.162]), while male fighters revealed −4.4% of percentage change (Δ_1_ = −3.2 (0.8) [−3.7, −2.7] kg; d_unb_ = −0.358 [−0.541, −0.222]). Blood glucose concentrations showed a significant increase in all subjects (Δ_1_ = 2.6 (0.9) [2.1, 3.0] mg/dL; d_unb_ = 0.424 [0.289, 0.591]), female (Δ_1_ = 2.0 (0.7) [1.3, 2.6] mg/dL; d_unb_ = 0.658 [0.311, 1.163]), and male (Δ_1_ = 3.0 (0.8) [2.5, 3.4] mg/dL; d_unb_ = 0.764 [0.470, 1.158]). The lipid profile variables (total cholesterol, TG, HDL-c, and LDL-c) were significantly higher than baseline in all participants, female and male; except for HDL-c in the men group, which had no significant change (Δ_1_ = 0.15 (0.6) [−0.2, 0.5] mg/dL; d_unb_ = 0.018 [−0.029, 0.067]). BUN increased significantly in comparison to the initial values in all, female and male participants; however, creatinine did not show changes in the female population (Δ_1_ = 0.002 (0.004) [−0.001, 0.006] mg/dL; d_unb_ = 0.070 [−0.032, 0.185]). Testosterone concentration decreased significantly and showed a large ES after RWL (Δ_1_ = −1.06 (0.2) [−1.1, −0.9] pg/mL; d_unb_ = −1.245 [−1.871, −0.787]). Similarly, the concentration of TSH was significantly lower than baseline in all fighters (Δ_1_ = −0.72 (0.2) [−0.8, −0.6] mIU/mL; d_unb_ = −2.304 [−3.204, −1.570]), including the women (Δ_1_ = −0.81 (0.2) [−1.0, −0.5] mIU/mL; d_unb_ = −2.022 [−3.554, −0.976]), and men (Δ_1_ = −0.67 (0.2) [−0.8, −0.5] mIU/mL; d_unb_ = −2.271 [−3.477, −1.358]). Regarding body composition and nutritional status, phase angle showed a significant reduction in all participants (Δ_1_ = −0.5 (0.3) [−0.6, −0.3] °; d_unb_ = −0.642 [−0.962, −0.363]), female (Δ_1_ = −0.2 (0.1) [−0.4, −0.1] °; d_unb_ = −0.482 [−0.875, −0.200]) and male (Δ_1_ = −0.6 (0.4) [−0.8, −0.3] °; d_unb_ = −0.761 [−1.245, −0.367]). Active tissue mass, TBW and ICW measurements were all significantly lower than baseline scores after RWL in all studied population; however, ECW showed a significant increase in all fighters, female (trivial clinical effect, 0.2 > ES) and male (medium ES).

#### 3.1.3. Rapid Weight Gain (Δ_2_)

**Figure 3:**
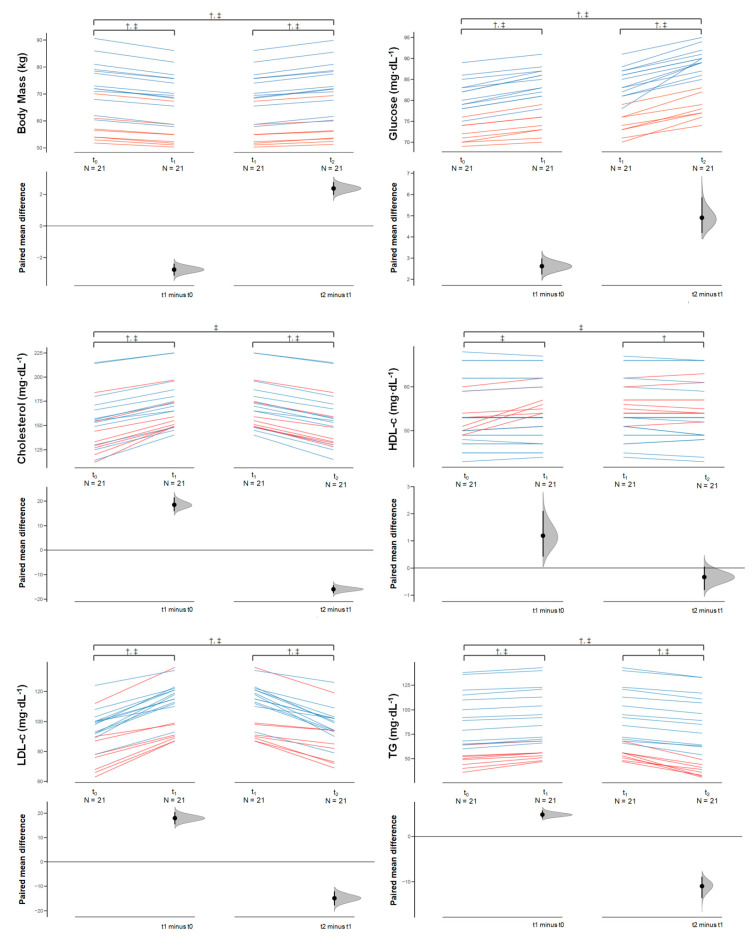

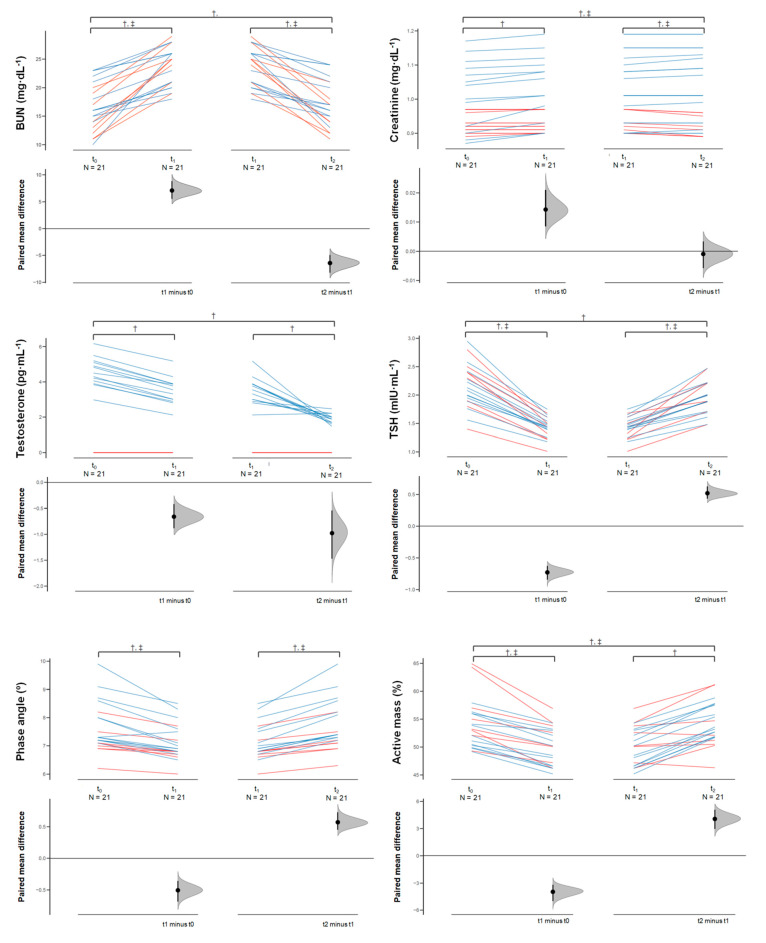

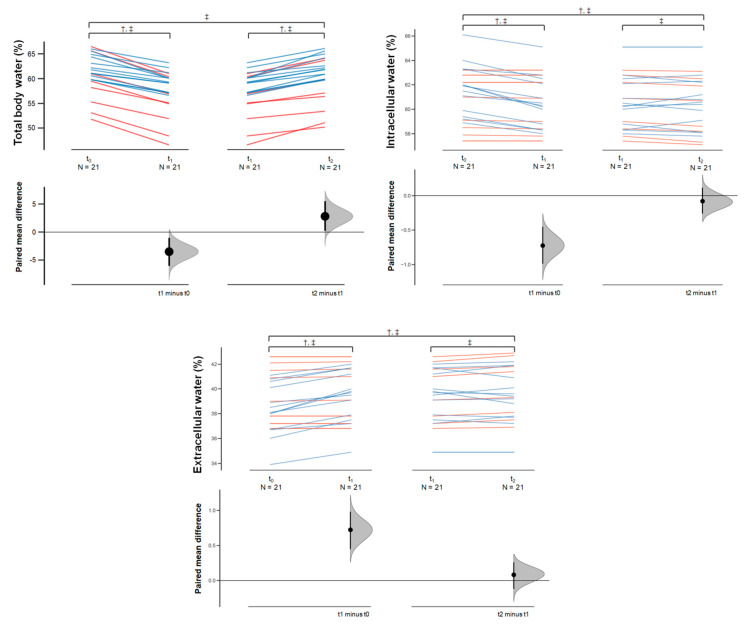
Multi-paired Cumming estimation plots of analyzed variables. All raw data are plotted on the upper ax. This displays the means and their 95% CIs across each timepoint measure (baseline [t_0_], after RWL [t_1_] and after RWG [t_2_]). The paired data are shown as lines (female = pink, male = cyan). In separate axes, beneath the raw data, the ES is shown with its 95% CI across three-time points for all participants (n = 21). Bootstrap resampling (BCa, 5000 bootstrap resamples) was performed to calculate the 95% CI of the mean difference. All confidence intervals were bias-corrected and accelerated with the DABEST ‘Data Analysis using Bootstrap-Coupled ESTimation’ software library within the R statistical computing environment. † Significance post-pre change in men (*p* < 0.05); ‡ Significance post-pre change in women (*p* < 0.05).

Almost all the reduced body mass was recovered after the RWG process (percentage of change = +3.6%) in all fighters (Δ_2_ = 2.3 (0.9) [1.9, 2.8] kg; d_unb_ = 0.204 [0.136, 0.286]); in particular, women had a significant increase of 2.5% (Δ_2_ = 1.4 (0.4) [1.0, 1.7] kg; d_unb_ = 0.221 [0.112, 0.384]), while men showed 4.2% increase after the RWG (Δ_2_ = 2.9 (0.7) [2.5, 3.4] kg; d_unb_ = 0.354 [0.205, 0.500]). Blood glucose concentrations augmented significantly in all participants (Δ_2_ = 4.9 (1.9) [4.0, 5.8] mg/dL; d_unb_ = 0.753 [0.505, 1.056]), female (Δ2 = 4.2 (1.2) [3.1, 5.3] mg/dL; d_unb_ = 1.271 [0.636, 2.217]) and male (Δ_2_ = 5.3 (2.2) [3.9, 6.6] mg/dL; d_unb_ = 1.547 [0.888, 2.400]). There was a significant reduction in total cholesterol, LDL-c and TG for all fighters, women and men, in comparison to baseline; however, HDL-c concentration only decreased significantly in men (Δ_2_ = −0.61 (0.9) [−1.1, −0.03] mg/dL; d_unb_ = −0.074 [−0.152, −0.004]), with no significant change in all participants (Δ_2_ = −0.33 (0.9) [−0.7, 0.1] mg/dL; d_unb_ = −0.049 [−0.115, 0.015]) and women (Δ_2_ = 0.12 (0.8) [−0.5, 0.8] mg/dL; d_unb_ = 0.030 [−0.125, 0.191]). BUN was significantly lower than baseline in all subjects, including female and male; notwithstanding, creatinine concentration decreased significantly in women (Δ_2_ = −0.01 (0.004) [−0.016, −0.008] mg/dL; d_unb_ = −0.353 [−0.622, 0.168]) while male participants had a significant increase (Δ_2_ = 0.006 (0.006) [0.002, 0.010] mg/dL; d_unb_ = 0.061 [0.019, 0.110]). Testosterone concentration continued decreasing after the RWG with a much larger ES than after RWL (Δ_2_ = −1.5 (0.9) [−2.1, −0.9] pg/mL; d_unb_ = −2.545 [−4.114, −1.277]). Conversely, TSH levels increased in all population (Δ_2_ = 0.51 (0.2) [0.4, 0.6] mIU/mL; d_unb_ = 2.093 [1.385, 2.851]), in both women and men groups. Whole phase angle, active tissue mass and TBW were significantly higher after RWG in all population; however, no significant change was found in active tissue mass for the female fighters (Δ_2_ = 2.0 (2.7) [−0.2, 4.3] %; d_unb_ = 0.392 [−0.045, 0.905]). ICW and ECW presented significant lower (Δ_2_ = −0.28 (0.1) [−0.3, −0.1] %; d_unb_ = 0.392 [−0.045, 0.905]) and higher values (Δ_2_ = 2.0 (2.7) [−0.2, 4.3] %; d_unb_ = 0.392 [−0.045, 0.905]) in women, respectively; but no significant changes were found in either all fighters or male participants. Figure 3 shows the multi-paired estimation plots for all analyzed variables.

#### 3.1.4. Overall Change (Δ_3_)

A significant decrease was found for changes in body mass in all participants (Δ_3_ = −0.3 (0.2) [−0.5, −0.2] kg; d_unb_ = −0.032 [−0.047, −0.019]) from the baseline to pre-competition (RWG), with a higher percentage of change in women (−1.0%) in comparison to men (−0.4%). Blood glucose concentration revealed a significant increase with large ES in all the fighters (Δ_3_ = 7.5 (2.2) [6.5, 8.5] mg/dL; d_unb_ = 1.190 [0.824, 1.645]). Total cholesterol and HDL-c concentration after RWG were significantly higher than baseline in women but not in men. LDL-c concentration augmented significantly in female and male, while the TG concentration was reduced in all individuals. Pre-competition BUN and creatinine values increased significantly in all participants when compared to baseline, except for female fighters who did not have a significant change in BUN (Δ_3_ = 0.2 (0.7) [−0.3, 0.8] mg/dL; dunb = 0.063 [−0.075, 0.212]). There was a large and significant decrease in testosterone concentration after the whole nutritional intervention (RWL and RWG) in the men group (Δ_3_ = −2.6 (1.0) [−3.2, −2.0] pg/mL; d_unb_ = −4.018 [−6.200, −2.346]). Women had no change in TSH concentration from the baseline to after RWG, but men had a significant reduction with a medium ES (Δ_3_ = −0.21 (0.1) [−0.2, −0.1] mIU/mL; d_unb_ = −0.622 [−0.985, −0.335]). Phase angle was fully recovered and showed no change after the intervention in all groups; however, after the regain process, women and men experienced significantly lower values of active tissue mass and ICW, while ECW was increased in all fighters. TBW had no change in men (Δ_3_ = 0.04 (0.2) [−0.2, 0.2] %; d_unb_ = 0.019 [−0.052, 0.0093]), but it decreased significantly in women (Δ_3_ = −1.9 (0.7) [−2.4, −1.3] %; d_unb_ = −0.315 [−0.555, −0.150]).

**Figure 4:**
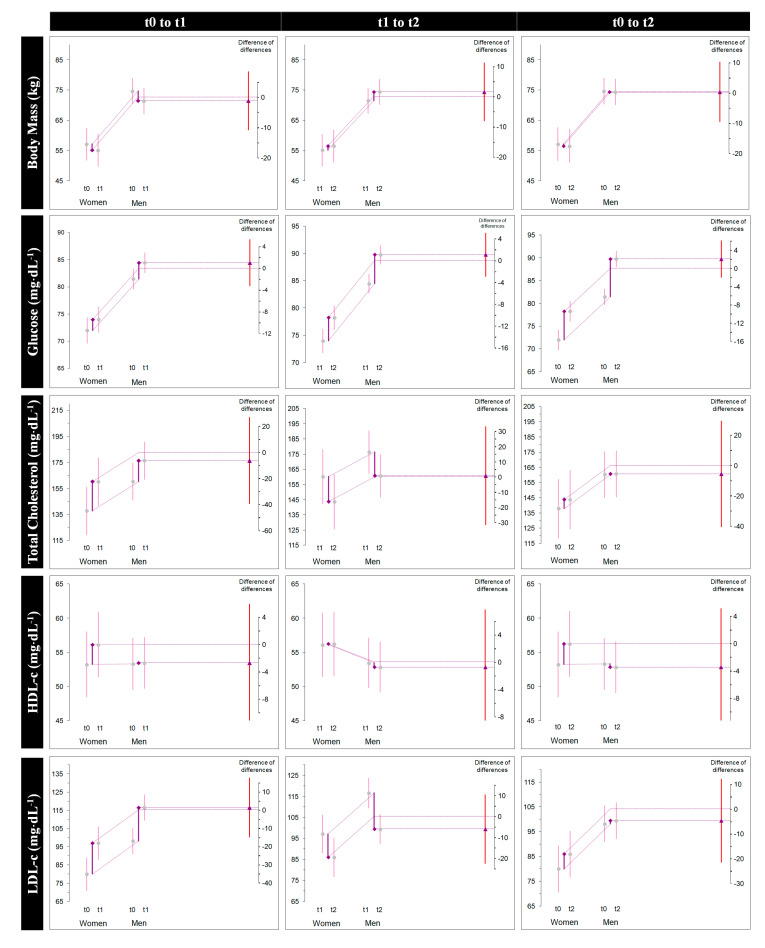

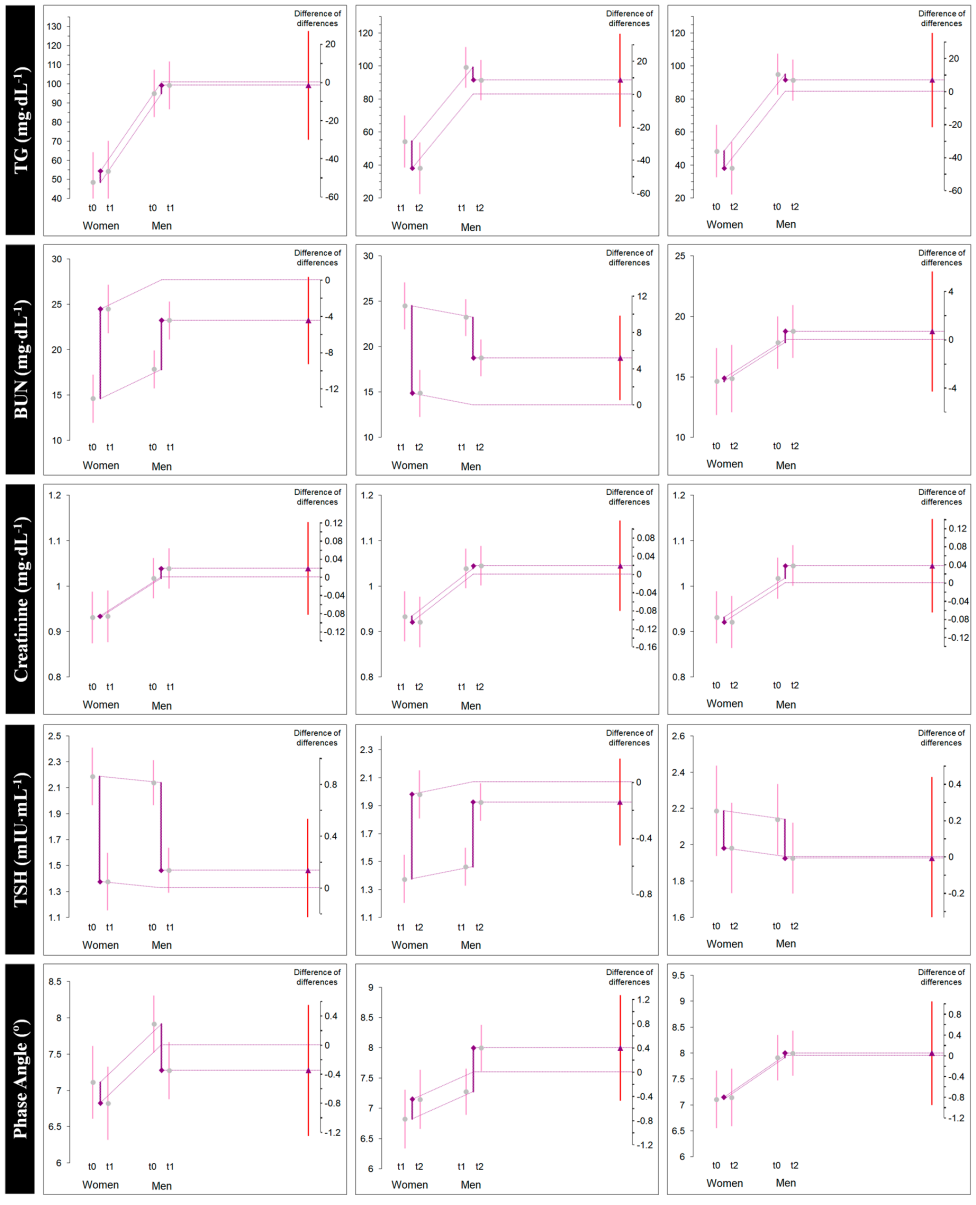

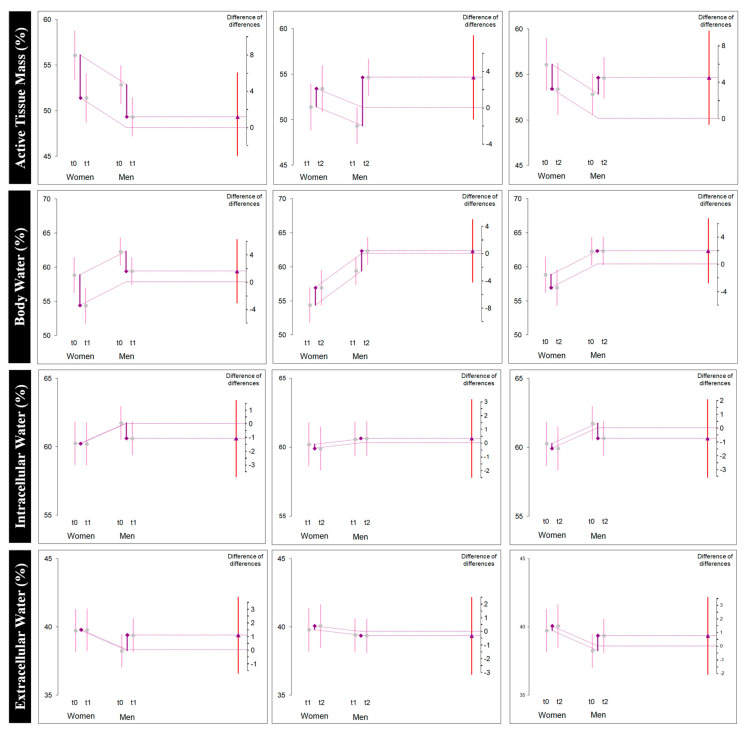
Difference-in-difference estimation plots for all variables. This graphic shows the difference of the differences, which is the calculation of the group means: (Male Δ—Female Δ). The effect chosen for examination is displayed as the triangle, with its 95% CI, against a floating different axis.

The independent between-sex analysis by Diff-in-Diff (Table 3) showed statistical difference solely in BUN concentration from RWL to after RWG (DID Δ_2_ = 5.16 [0.51, 9.80], *p* < 0.05). There was a trend to significance difference for men and women in BUN after the RWL compared to baseline (DID Δ_1_ = −4.49 [−9.28, 0.30], *p* = 0.066), with no important magnitude of change before and after the overall intervention. Although not significant, the change from baseline to after RWG in active tissue mass was different in magnitude and sense (Δ_3_ = −2.6 (0.6) [−3.2, −2.0] %; d_unb_ = −0.432 [−0.749, −0.222] vs. Δ_3_ = 1.8 (0.5) [1.4, 2.1] %; d_unb_ = 0.604 [0.639, 0.917]) between female and male fighters, respectively (DID Δ_3_ = 4.48 [−0.68, 9.64], *p* = 0.08). All other variables did not reveal significant or important sex differences (Figure 4).

## 4. Discussion

The aim of this clinical study was to assess the acute effects of a supervised RWL and RWG nutrition intervention of “making weight” on health markers, hormone levels, and body composition in Muay Thai fighters. According to our initial hypothesis, we found that a supervised nutritional process reduced the drastic impact of RWL and RWG on body mass change, while all athletes made the weight. As a result of glycogen depletion and emptying the intestinal content, a ~3% reduction in body mass was expected in a short duration (1 to 7 days) cutting process [8]. In this study, the percentage of change after the RWL intervention was between 3.5% and 4.4% for women and men, respectively. It has been shown previously that reductions in body mass between 5.3–9.1% have deleterious effects on muscle and cognitive performance [25], which is common within the unsupervised practices in MMA and sports with weight classes, including combat sports [2,26]. Actually, there is a higher risk of adverse effects when the loss is higher than 5% of body mass, and athletes in the highest percentiles of RWL (reductions between 8–10% in body mass after RWL) may be at greater risk of an adverse event, such as decrement in physical performance, feeling fatigue or weakness, dizziness, feverishness, nausea, or cramps, among others [8]. Interestingly, our study demonstrated that the male fighters recovered almost all the reduced body mass with only 8 h of RWG (4.2%), although women presented certain difficulty to regain body mass (2.5%). The entire process of RWL and RWG resulted in a significant decrease in body mass in both women (−1.0%) and men (−0.4%) at pre-competition, but it is important to highlight that the RWG seems to be more relevant for MMA success in a real-life competition [27]. On the other hand, it has been demonstrated that the consumption of a high-carbohydrate intake during a short regain period guarantees that most of the depleted glycogen is restored, especially after a low-carbohydrate diet [8,19]. In this regard, our results showed a significantly higher glucose availability in all participants throughout the intervention (Δ1, Δ2, and Δ3), with no significant difference in the change between men and women (*p* > 0.05). These findings are contrary to previous unsupervised practices in observational studies [28,29]. The lower concentration of blood glucose that is frequently seen after the RWL has been associated to the increase in serum cortisol [28], which provides a partial explanation for concomitant hormonal changes and side effects during this drastic body mass reduction (>5% in body mass) that is not seen in a normal weight loss group.

Previous studies have shown that body mass reduction may lead to alterations in lipid profile, with a frequent reduction in TG [30]; however, the results are debatable because of the different regimes used across the studies and the multiple factors that are not only related to diet (e.g., physical exercise). For example, in Korean wrestlers, a significant decrease in total cholesterol, LDL-c, glucose, and superoxide dismutase has been reported after a short-term weight reduction intervention [31]. Notwithstanding, the effect of RWL on lipid markers depends on the magnitude of body mass reduction, as it has been reported for judo athletes after evaluation of the prooxidative-antioxidative system diversity [32]. In our study, total cholesterol, LDL, and TG concentrations showed similar behavior during the RWL and RWG process (increase and reduction, respectively), although all the concentrations were within the normal range at pre-competition. HDL-c concentration after RWG was significantly higher than baseline in women but not in men (no between-sex statistical difference was found). The reduction in TG concentration, and possible increase in FFA and glycerol (not measured), might be the consequence of the increased lipolysis in adipose tissue and the hormonal adaptations induced by the RWL and RWG (i.e., low testosterone, increase in the sensitivity to cortisol, increase in cortisol secretion) which improves lipid utilization [33]. On the other hand, regarding renal markers, the significant difference between female and male showed a higher susceptibility to changes in BUN concentration in women, nonetheless both BUN and creatinine were within normal values in all studied participants. Creatinine can be useful to differentiate prerenal from renal causes when the BUN is increased [34]; however, our study did not reveal important changes in the BUN-to-creatinine ratio, since the average values of this index were among the normal range (15–20) in all fighters (data not shown). We highlight the fact that there are more sensitive and specific markers to evaluate acute kidney injury than BUN and serum creatinine [35], given that recent evidence suggest that the BUN-to-creatinine ratio is not a reliable parameter for distinguishing prerenal from intrinsic acute kidney injury [36]. Our findings did not reveal negative impact on kidney health, contrary to what has been reported in participants that abuse of dehydration methods and diuretics to making weight [15,37], but further research is needed with more sensitive markers and long term monitoring to assess properly the impact of RWL and RWG.

Negative hormonal changes have been found in previous studies after an RWL process in combat sports [28,38,39]. Degoutte et al. [40] reported a significant decrease in testosterone concentration in judo athletes that underwent the combination of energy restriction and intense exercise training for an RWL before a competition. These authors concluded that drastic body mass reduction before a competition adversely affects the physiology and psychology of the fighters and impairs physical performance. Our findings are in agreement with preceding investigations in this regard (except for psychological variables which were not measured in this study). We found a significant and clinically important (very large ES) decrease in testosterone concentration in men after RWL, with no restoration and continuous reduction even after the RWG process. These findings highlight the catabolic hormonal responses that occurred during the energy restriction, which were maintained after the regain process; therefore, further studies are required to evaluate the time course of testosterone concentration and its impact on performance. Similarly, male fighters had a significant reduction in TSH concentration after the RWL and RWG intervention, but no change was found in women at pre-competition. This hormonal response may be due to the strong influence of caloric restriction on the hypothalamic-pituitary-thyroid axis [41]. Furthermore, in order to return to the baseline, TSH values needed 15 days in men and 25 days in some women (data not shown). Hence, although an accurate and individualized nutritional intervention has been implemented in our study, an impact on the hormonal profile was inevitable. Importantly, some psychological variables, such as fighting motivation, mental toughness, competition anxiety, self-efficacy, and self-confidence, represent cause-and-effect variables of the relationship between hormonal changes and combat performance, as it has been reported previously [42,43,44].

Various parameters of body composition and nutritional status were measured by BIA analysis. In particular, phase angle showed no change after the whole RWL and RWG intervention, which indicates a total recovery of cellular integrity at pre-competition. However, active tissue mass was reduced significantly after the intervention (less magnitude of recovery of this variable was seen in female fighters after the RWL). Moreover, the change in this variable was different in magnitude and sense between sexes (Figure 4). This reduction in what represents the metabolically active portion of the body is in agreement with the recent findings of Roklicer et al. [45], who reported skeletal muscle damage to a significant extent in male jūdōkas who underwent a three-day RWL and strenuous exercise. Further research is needed to analyze the impact of RWL and RWG on muscle mass between males and females. As expected, hydration status variables such as TBW and ICW decreased significantly after the RWL, with a concomitant increase in ECW; however, after the regain process, only females experienced a significant reduction in TBW and ICW while ECW was increased. Previous studies on hydration status have found that even experienced and elite fighters are not successfully rehydrated, either in the evening before the fight or in the weigh-in close to competition [46,47].

## 5. Practical Applications

If implemented, we recommend operating the making weight process on a scientific-based and individual-response methodology that considers a body mass reduction that does not exceed 5%. With regards to sex differences, it is advisable to perform an RWL and RWG mainly, if not exclusively, on male athletes (avoid it if the female athlete already shows irregularities in the menstrual cycle or previous episodes of amenorrhea). Moreover, in any case, this weight-cutting should not be used more than 2–3 times a year, given that performing this process several times and close between each other might cause permanent changes to the hypothalamic-pituitary-thyroid axis. In agreement with De Crée [48], we recommend to exercise and nutrition professionals to (i) not encourage fighters to engage in cycling, continuous and/or mandatory weight cutting; (ii) be aware of the effects of RWL and RWG at the physiological and performance level; (iii) be aware of the sex differences that might occur with the hormonal responses and hydration status; (iv) be aware of the potential nutritional strategies (including nutritional supplementation) to reduce the adverse events after an RWL and RWG practice.

## 6. Limitations

Our study has several limitations that should be mentioned: (i) muscle glycogen concentration was not measure, so we cannot determine if a significant depletion and complete restoration happened; notwithstanding, several previous studies support this physiological response to low- and high-carbohydrate diet during a RWL and RWG process [8,19]; (ii) there are more sensitive and specific markers than BUN and serum creatinine in order to evaluate acute kidney injury, which makes necessary future research on inflammatory markers that are affected after low-carbohydrate diets (e.g., microRNAs and in vitro) [49,50]; (iii) we did not measure sports performance variables to infer if the supervised RWL and RWG strategy was enough to counteract the frequent adverse effects of the making weight process at the performance and psychological level; (iv) we are aware of the limitations but also the advantages of BIA to assess body composition. Actually, bioelectrical impedance vector analysis (BIVA) has emerged as an alternative technique to overcome conventional BIA limitations, considering the use of raw impedance variables. However, given that only phase angle was reported as raw data, we could infer that a partial evaluation based on BIVA was performed (impedance ratio was missing). In addition, the ImpediMed DF50 device requires more validation for this regard; (v) the question about frequency of making weight remains unclear, but we have shown a negative impact on the hormonal axis. Moreover, it would be very interesting to design a large-scale study for a period that covers at least an entire competitive season (at least for a year), involving a larger number of participants and evaluating a wider hormonal panel (including the whole profile of the pituitary gland, cortisol, and thyroid hormones) and, inflammatory and antioxidant markers such as miRNAs.

## 7. Conclusions

We have demonstrated that making weight in high-level Muay Thai fighters might be effective and less dangerous if performed under the careful supervision of professionals with experience, not only in sports nutrition, but also in combat sports and weight cutting. Thus, an evidence-based and individualized nutrition methodology reduces the adverse events after an RWL and RWG practice, although the impact on the hormonal profile is inevitable. Further research is necessary to prevent acute dehydration and the sex-dependent responses on female fighters.

## Figures and Tables

**Table 1:** Descriptive information of participants at baseline.

Variable	$\bar{x}$ (SD) (Women *n* = 8)	95% CI (min, max)	$\bar{x}$ (SD) (Men *n* = 13)	95% CI (min, max)
Age (years)	24.62 (5.42)	20.09, 29.15	26.53 (6.35)	22.69, 30.38
Stature (m)	163.00 (3.11)	160.39, 165.60	177.07 (4.80)	174.17, 179.97
Body mass (kg)	57.17 (5.96)	52.19, 62.15	74.71 (8.68)	69.46, 79.96
BMI (kg∙m^−2^)	21.54 (2.51)	19.44, 23.65	22.74 (1.89)	21.60, 23.89
Glucose (mg/dL)	72.00 (2.44)	69.95, 74.04	81.46 (3.82)	79.15, 83.77
Total Cholesterol (mg/dL)	137.87 (22.78)	118.82, 156.92	160.38 (30.21)	142.12, 178.64
HDL-c (mg/dL)	53.25 (4.20)	49.73, 56.76	53.30 (8.00)	48.47, 58.14
LDL-c (mg/dL)	80.00 (16.13)	66.51, 93.48	98.15 (10.76)	91.64, 104.66
TG (mg/dL)	48.50 (8.65)	41.26, 49.50	95.07 (27.26)	78.60, 111.55
BUN (mg/dL)	14.62 (3.58)	11.62, 17.62	17.84 (4.18)	15.32, 20.37
Creatinine (mg/dL)	0.93 (0.03)	0.90, 0.95	1.01 (0.10)	0.95, 1.07
Testosterone (pg/mL)	No measure	No measure	4.57 (0.82)	4.07, 5.07
TSH (mIU/mL)	2.18 (0.45)	1.80, 2.56	2.14 (0.36)	1.92, 2.35
Whole-body phase angle (°)	7.11 (0.56)	6.63, 7.58	7.91 (0.90)	7.36, 8.46
Body fat (%)	17.73 (2.05)	16.02, 19.45	11.46 (1.30)	10.67, 12.25
Active tissue mass (%)	56.10 (5.69)	51.33, 60.86	52.85 (2.98)	51.05, 54.65
Total body water (%)	58.87 (5.41)	54.35, 63.39	62.31 (2.26)	60.94, 63.68
Intracellular water (%)	60.26 (2.31)	58.32, 62.20	61.73 (2.10)	60.46, 63.00
Extracellular water (%)	39.73 (2.31)	37.79, 41.67	38.26 (2.10)	36.99, 39.53

Data are presented as mean ± standard deviation. The coefficient of variation for all variables ranged from 0 to 5.0%. Repeat assays in our laboratory have revealed a coefficient of variation of less than 5.0% with the Advia analyzers and less than 3.0% with the ImpediMED DF50 device. BM: body mass; BMI: body mass index; BUN, blood urea nitrogen; HDL-c: high-density lipoprotein cholesterol; LDL: low-density lipoprotein cholesterol; TG: triacylglycerol; TSH: thyroid-stimulating hormone.

**Table 2:** Repeated measures across the three timepoints.

		Baseline	RWL	RWG	Comparison of the Means					
Variable	Group	t0 $\bar{x}$ (SD)	t1 $\bar{x}$(SD)	t2 $\bar{x}$ (SD)	Δ_1_ $\bar{x}$ (SD) [95% CI]	d_unb_ δ [95% CI]	Δ_2_ $\bar{x}$ (SD) [95% CI]	d_unb_ δ [95% CI]	Δ_3_ $\bar{x}$ (SD) [95% CI]	d_unb_ δ [95% CI]
BM (kg)	All	68.0 (11.5)	65.2 (10.8)	67.6 (11.6)	−2.7 (0.9) [−3.1, −2.3] *	−0.237 [−0.329, −0.163]	2.3 (0.9) [1.9, 2.8] *	0.204 [0.136, 0.286]	−0.3 (0.2) [−0.5, −0.2] *	−0.032 [−0.047, −0.019]
	Female	57.1 (5.9)	55.1 (5.6)	56.5 (5.8)	−2.0 (0.4) [−2.3, −1.6] *	−0.309 [−0.533, −0.162]	1.4 (0.4) [1.0, 1.7] *	0.221 [0.112, 0.384]	−0.5 (0.2) [−0.7, −0.4] *	−0.088 [−0.155, −0.042]
	Male	74.7 (8.6)	71.4 (8.1)	74.4 (8.5)	−3.2 (0.8) [−3.7, −2.7] *	−0.358 [−0.541, −0.222]	2.9 (0.7) [2.5, 3.4] *	0.354 [0.205, 0.500]	−0.2 (0.1) [−0.3, −0.1] *	−0.028 [−0.047, −0.013]
Glucose (mg/dL^−1^)	All	77.8 (5.7)	80.4 (6.1)	85.3 (6.3)	2.6 (0.9) [2.1, 3.0] *	0.424 [0.289, 0.591]	4.9 (1.9) [4.0, 5.8] *	0.753 [0.505, 1.056]	7.5 (2.2) [6.5, 8.5] *	1.190 [0.824, 1.645]
	Female	72.0 (2.4)	74.0 (2.9)	78.2 (3.0)	2.0 (0.7) [1.3, 2.6] *	0.658 [0.311, 1.163]	4.2 (1.2) [3.1, 5.3] *	1.271 [0.636, 2.217]	6.2 (1.3) [5.0, 7.4] *	2.022 [1.058, 3.489]
	Male	81.4 (3.8)	84.4 (3.5)	89.7 (2.8)	3.0 (0.8) [2.5, 3.4] *	0.764 [0.470, 1.158]	5.3 (2.2) [3.9, 6.6] *	1.547 [0.888, 2.400]	8.3 (2.2) [6.9, 9.6] *	2.303 [0.672, 3.981]
Total Cholesterol (mg/dL)	All	151.8 (29.2)	170.2 (24.5)	154.2 (27.3)	18.4 (6.4) [15.5, 21.4] *	0.659 [0.449, 0.917]	−16.0 (3.8) [−17.7, −14.2] *	−0593 [−0.815, −0.416]	2.4 (4.0) [0.6, 4.3] *	0.084 [0.020, 0.153]
	Female	137.8 (22.7)	160.2 (17.1)	143.7 (19.3)	22.3 (7.6) [15.9, 28.7] *	0.986 [0.479, 1.730]	−16.5 (3.3) [−19.3, −13.6] *	−0.802 [−1.381, −0.423]	5.8 (4.9) [1.7, 10.0] *	0.247 [0.057, 0.488]
	Male	160.3 (30.2)	176.4 (26.8)	160.7 (30.1)	16.0 (4.3) [13.4, 18.7] *	0.526 [0.324, 0.798]	−15.6 (4.2) [−18.2, −13.1] *	−0.515 [−0.780, −0.317]	0.38 (0.65) [−0.008, 0.77] ^NS^	0.012 [0.000, 0.025]
HDL-c (mg/dL)	All	53.2 (6.6)	54.4 (6.5)	54.1 (6.6)	1.9 (2.0) [0.27, 2.10] *	0.173 [0.036, 0.320]	−0.33 (0.9) [−0.77, 0.10] ^NS^	−0.049 [−0.115, 0.015]	0.85 (2.2) [−0.15, 1.86] ^NS^	0.124 [−0.021, 0.275]
	Female	53.2 (4.2)	56.1 (3.5)	56.2 (3.8)	2.8 (2.3) [0.9, 4.8] *	0.659 [0.160, 1.296]	0.12 (0.83) [−0.57, 0.82] ^NS^	0.030 [−0.125, 0.191]	3.0 (2.1) [1.2, 4.7] *	0.662 [0.203, 1.264]
	Male	53.3 (8.0)	53.4 (7.8)	52.8 (7.7)	0.15 (0.68) [−0.26, 0.57] ^NS^	0.018 [−0.029, 0.067]	−0.61 (0.96) [−1.19, −0.03] *	−0.074 [−0.152, −0.004]	−0.46 (0.7) [−0.93, 0.007] ^NS^	−0.055 [−0.116, 0.001]
LDL-c (mg/dL)	All	91.2 (15.5)	109.1 (15.5)	94.3 (14.5)	17.9 (5.9) [15.2, 20.6] *	1.111 [0.762, 1.543]	−14.8 (6.7) [−17.9, −11.7] *	−0.951 [−1.344, −0.625]	3.09 (2.5) [1.9, 4.2] *	0.198 [0.108, 0.300]
	Female	80.0 (16.1)	97.1 (16.3)	86.0 (16.4)	17.1 (6.1) [12.02, 22.2] *	0.936 [0.450, 1.647]	−11,1 (5.9) [−16.1, −6.1] *	−0.603 [−1.101, −0.242]	6.0 (1.3) [4.9, 7.0] *	0.327 [0.172, 0.565]
	Male	98.1 (10.7)	116.6 (9.3)	99.46 (10.9)	18.4 (5.9) [14.8, 22.0] *	1.710 [1.031, 2.611]	−17.1 (6.3) [−20.9, −13.3] *	−1.574 [−2.420, −0.931]	1.3 (0.8) [0.7, 1.8] *	0.113 [0.055, 0.183]
TG (mg/dL)	All	77.3 (31.7)	82.1 (30.9)	71.1 (34.1)	4.8 (2.0) [3.9, 5.7] *	0.149 [0.099, 0.210]	−11.0 (5.3) [−13.4, −8.5] *	−0.325 [−0.462, −0.211]	−6.1 (5.5) [−8.6, −3.6] *	−0.179 [−0.277, −0.093]
	Female	48.5 (8.6)	54.3 (6.5)	38.1 (6.3)	5.8 (2.6) [3.6, 8.1] *	0.679 [0.297, 1.220]	−16.2 (5.0) [−20.4, −12.0] *	−2.237 [−3.908, −1.111]	−10.3 (7.1) [−16.3, −4.3] *	−1.215 [−2.307, −0.387]
	Male	95.0 (27.2)	99.3 (27.0)	91.5 (27.1)	4.2 (1.2) [3.4, 4.9] *	0.146 [0.089, 0.221]	−7.7 (1.9) [−8.9, −6.5] *	−0.268 [−0.405, −0.166]	−3.5 (1.1) [−4.2, −2.8] *	−0.122 [−0.186, −0.074]
BUN (mg/dL)	All	16.6 (4.1)	23.7 (3.4)	17.28 (4.1)	7.0 (3.7) [5.3, 8.8] *	1.781 [1.134, 2.552]	−6.4 (3.8) [−8.1, −4.6] *	−1.626 [−2.356, −1.007]	0.6 (0.8) [0.2, 1.0] *	0.154 [0.058, 0.259]
	Female	14.6 (3.5)	24.5 (3.2)	14.8 (3.4)	9.8 (2.4) [7.8, 11.9] *	2.548 [1.313, 4.413]	−9.6 (2.8) [−11.9, −7.2] *	−2.522 [−4.393, −1.268]	0.2 (0.7) [−0.3. 0.8] ^NS^	0.063 [−0.075, 0.212]
	Male	17.8 (4.1)	23.2 (3.5)	18.7 (3.8)	5.3 (3.4) [3.2, 7.4] *	1.298 [0.643, 2.105]	−4.4 (2.9) [−6.2, −2.6] *	−1.118 [−1.821, −0.547]	0.9 (0.8) [0.4, 1.4] *	0.214 [0.078, 0.372]
Creatinine (mg/dL)	All	0.98 (0.09)	0.99 (0.09)	0.99 (0.09)	0.01 (0.01) [0.007, 0.020] *	0.151 [0.071, 0.239]	−0.01 (0.01) [−0.005, 0.004] ^NS^	−0.010 [−0.059, 0.039]	0.01 (0.02) [0.003, 0.020] *	0.136 [0.028, 0.251]
	Female	0.93 (0.03)	0.93 (0.03)	0.92 (0.03)	0.002 (0.004) [−0.001, 0.006] ^NS^	0.070 [−0.032, 0.185]	−0.01 (0.004) [−0.016, −0.008] *	−0.353 [−0.622, −0.168]	−0.01 (0.005) [−0.01, −0.005] *	−0.281 [−0.513, −0.113]
	Male	1.01 (0.10)	1.03 (0.09)	1.04 (0.09)	0.02 (0.01) [0.013, 0.030] *	0.208 [0.102, 0.338]	0.006 (0.006) [0.002, 0.010] *	0.061 [0.019, 0.110]	0.02 (0.01) [0.018, 0.037] *	0.265 [0.139, 0.424]
Testosterone (pg/mL)	Male	4.57 (0.82)	3.50 (0.77)	1.92 (0.27)	−1.06 (0.2) [−1.1, −0.9] *	−1.245 [−1.871, −0.787]	−1.5 (0.9) [−2.1, −0.9] *	−2.545 [−4.114, −1.277]	−2.6 (1.0) [−3.2, −2.0] *	−4.018 [−6.200, −2.346]
TSH (mIU/mL)	All	2.15 (0.38)	1.43 (0.18)	1.94 (0.28)	−0.72 (0.2) [−0.8, −0.6] *	−2.304 [−3.208, −1.570]	0.51 (0.2) [0.4, 0.6] *	2.093 [1.385, 2.951]	−0.21 (0.2) [−0.3, −0.1] *	−0.595 [−0.970, −0.255]
	Female	2.18 (0.45)	1.37 (0.22)	1.98 (0.31)	−0.81 (0.2) [−1.0, −0.5] *	−2.022 [−3.554, −0.976]	0.6 (0.3) [0.3, 0.8] *	1.974 [0.819, 3.582]	−0.20 (0.3) [−0.5, 0.1] ^NS^	−0.468 [−1.236, 0.214]
	Male	2.14 (0.36)	1.46 (0.15)	1.92 (0.27)	−0.67 (0.2) [−0.8, −0.5] *	−2.271 [−3.477, −1.358]	0.46 (0.1) [0.3, 0.5] *	1.964 [1.187, 2.996]	−0.21 (0.1) [−0.2, −0.1] *	−0.622 [−0.985, −0.335]
Whole body phase angle (°)	All	7.60 (0.87)	7.10 (0.61)	7.67 (0.84)	−0.5 (0.3) [−0.6, −0.3] *	−0.642 [−0.962, −0.363]	0.5 (0.3) [0.4, 0.7] *	0.739 [0.464, 1.066]	0.06 (0.27) [−0.05, 0.19] ^NS^	0.074 [−0.061, 0.214]
	Female	7.11 (0.56)	6.82 (0.48)	7.15 (0.54)	−0.2 (0.1) [−0.4, −0.1] *	−0.482 [−0.875, −0.200]	0.3 (0.08) [0.2, 0.3] *	0.559 [0.285, 0.971]	0.03 (0.14) [−0.08, 0.15] ^NS^	0.060 [−0.113, 0.243]
	Male	7.91 (0.90)	7.27 (0.64)	8.00 (0.85)	−0.6 (0.4) [−0.8, −0.3] *	−0.761 [−1.245, −0.367]	0.7 (0.3) [0.5, 0.9] *	0.893 [0.506, 1.392]	0.08 (0.33) [−0.11, 0.28] ^NS^	0.090 [−0.116, 0.304]
Active tissue mass (%)	All	54.0 (4.3)	50.1 (3.4)	54.1 (3.7)	−3.9 (2.1) [−4.9, −2.9] *	−0.969 [−1.390, −0.616]	4.0 (2.4) [2.9, 5.1] *	1.085 [0.667, 1.577]	0.1 (2.3) [−0.9, 1.1] ^NS^	0.023 [−0.216, 0.265]
	Female	56.1 (5.6)	51.4 (3.6)	53.4 (5.3)	−4.6 (3.1) [−7.2, −2.0] *	−0.870 [−1.640, −0.290]	2.0 (2.7) [−0.2, 4.3] ^NS^	0.392 [−0.045, 0.905]	−2.6 (0.6) [−3.2, −2.0] *	−0.432 [−0.749, −0.222]
	Male	52.8 (2.9)	49.3 (3.1)	54.6 (2.6)	−3.5 (1.1) [−4.2, −2.8] *	−1.076 [−1.644, −0.647]	5.3 (1.1) [4.6, 6.0] *	1.728 [1.083, 2.603]	1.8 (0.5) [1.4, 2.1] *	0.604 [0.639, 0.917]
Body water (%)	All	61.0 (4.0)	57.5 (4.2)	60.3 (4.4)	−3.4 (1.2) [−4.0, −2.9] *	−0.810 [−1.128, −0.551]	2.8 (1.0) [2.3, 3.2] *	0.616 [0.416, 0.861]	−0.7 (1.0) [−1.2, −0.2] *	−0.157 [−0.281, −0.042]
	Female	58.8 (5.4)	54.4 (5.2)	56.9 (5.3)	−4.4 (0.8) [−5.2, −3.7] *	−0.749 [−1.288, −0.397]	2.5 (1.0) [1.6, 3.4] *	0.435 [0.198, 0.774]	−1.9 (0.7) [−2.4, −1.3] *	−0.315 [−0.555, −0.150]
	Male	62.3 (2.2)	59.4 (2.0)	62.3 (2.2)	−2.8 (1.0) [−3.5, −2.2] *	−1.256 [−1.928, −0.746]	2.9 (1.0) [2.3, 3.5] *	1.291 [0.766, 1.982]	0.04 (0.29) [−0.1, 0.2] ^NS^	0.019 [−0.052, 0.093]
Intracellular water (%)	All	61.1 (2.2)	60.4 (2.1)	60.3 (2.2)	−0.72 (0.6) [−1.0, −0.4] *	−0.317 [−0.487, −0.167]	−0.08 (0.4) [−0.2, 0.1] ^NS^	−0.036 [−0.126, 0.053]	−0.8 (0.4) [−1.01, −0.5] *	−0.347 [−0.502, −0.217]
	Female	60.2 (2.3)	60.2 (2.3)	59.9 (2.4)	−0.05 (0.05) [−0.09, −0.005] *	−0.019 [−0.040, −0.002]	−0.28 (0.1) [−0.3, −0.1] *	−0.107 [−0.192, −0.048]	−0.3 (0.1) [−0.4, −0.2] *	−0.127 [−0.227, −0.056]
	Male	61.7 (2.1)	60.6 (2.0)	60.6 (2.1)	−1.13 (0.4) [−1.39, −0.88] *	−0.509 [−0.783, −0.300]	0.046 (0.5) [−0.2, 0.3] ^NS^	0.021 [−0.117, 0.160]	−1.0 (0.3) [−1.2, −0.8] *	−0.484 [−0.737, −0.294]
Extracellular water (%)	All	38.8 (2.2)	39.5 (2.1)	39.6 (2.2)	0.7 (0.6) [0.4, 1.0] *	0.317 [0.167, 0.487]	0.08 (0.45) [−0.1, 0.2] ^NS^	0.036 [−0.053, 0.126]	0.80 (0.46) [0.5, 1.0] *	0.347 [0.217, 0.502]
	Female	39.7 (2.3)	39.7 (2.3)	40.0 (2.4)	0.05 (0.05) [0.005, 0.09] *	0.019 [0.002, 0.040]	0.28 (0.12) [0.1, 0.3] *	0.107 [0.048, 0.192]	0.33 (0.15) [0.2, 0.4] *	0.127 [0.056, 0.227]
	Male	38.2 (2.1)	39.4 (2.0)	39.3 (2.1)	1.1 (0.4) [0.8, 1.3] *	0.509 [0.300, 0.783]	−0.04 (0.5) [−0.3, 0.2] ^NS^	−0.021 [−0.160, 0.117]	1.09 (0.33) [0.8, 1.2] *	0.484 [0.294, 0.737]

Data is presented as mean ($\bar{x}$) and standard deviation (SD). * Statistically significant change (*p* < 0.05); ^NS^ No significant change.

**Table 3:** Difference of differences between male and female.

	Diff-In-Diff from t_0_ to t_1_ (Δ_1_)				Diff-In-Diff from t_1_ to t_2_ (Δ_2_)				Diff-In-Diff from t_0_ to t_2_ (Δ_3_)			
Variable	Mean (Male Δ_1_—Female Δ_1_	DID	95% CI	*p*	Mean (Male Δ_2_—Female Δ_2_	DID	95% CI	*p*	Mean (Male Δ_3_—Female Δ_3_	DID	95% CI	*p*
BM (kg)	−3.22–−2.01	−1.21	−10.94, 8.52	0.803	2.96–1.42	1.53	−8.12, 11.2	0.749	−0.26–−0.58	0.32	−9.63, 10.28	0.948
Glucose (mg/dL)	3.00–2.00	1.00	−3.31, 5.30	0.641	5.30–4.25	1.05	−2.96, 5.07	0.597	8.30–6.25	2.05	−2.00, 6.12	0.312
Total Cholesterol (mg/dL)	16.07–22.37	−6.29	−39.50, 26.90	0.703	−15.69–−16.50	0.80	−31.68, 33.29	0.960	0.38–5.87	−5.49	−40.47, 29.49	0.752
HDL-c (mg/dL)	0.15–2.87	−2.72	−11.36, 5.91	0.527	−0.61–−0.12	−0.74	−9.19, 7.71	0.860	−0.46–3.00	−3.46	−12.10, 5.17	0.422
LDL-c (mg/dL)	18.46–17.125	1.33	−15.03, 17.70	0.870	−17.15–−11.13	−6.02	−22.54, 10.48	0.464	1.30–6.00	−4.69	−21.57, 12.18	0.577
TG (mg/dL)	4.23–5.87	−1.64	−30.07, 26.78	0.907	−7.76–−16.25	8.48	−19.72, 36.68	0.546	−3.53–−10.38	6.83	−21.62, 35.29	0.630
BUN (mg/dL)	5.38–9.87	−4.49	−9.28, 0.30	0.066	−4.46–−9.62	5.16 *	0.51, 9.80	0.03	0.92–0.25	0.67	−4.29, 5.64	0.785
Creatinine (mg/dL)	0.02–0.0025	0.019	−0.08, 0.12	0.709	0.006–−0.013	0.018	−0.08, 0.11	0.706	0.027–−0.01	0.037	−0.06, 0.14	0.463
TSH (mIU/mL)	−0.67–−0.81	−0.13	−0.26, 0.53	0.496	0.46–0.60	−0.143	−0.45, 0.16	0.356	−0.213–−0.205	−0.008	−0.45, 0.43	0.971
Whole body phase angle (°)	−0.63–−0.28	−0.35	−1.25, 0.55	0.436	0.72–0.32	−0.398	−0.47, 1.27	0.362	0.084–0.037	0.047	−0.95, 1.04	0.924
Active tissue mass (%)	−3.52–−4.68	1.16	−3.71, 6.04	0.632	5.33–2.01	3.318	−1.30, 7.94	0.154	1.80–2.67	4.482	−0.68, 9.64	0.087
Body water (%)	−2.89–−4.47	1.58	−3.11, 6.27	0.499	2.93–2.57	0.36	−4.28, 5.02	0.875	0.046–−1.900	1.946	−2.85, 6.71	0.414
Intracellular water (%)	−1.13–−0.05	−1.08	−3.89, 1.72	0.438	0.046–−0.28	0.333	−2.51, 3.17	0.813	−1.092–−0.337	−0.755	−3.59, 2.08	0.594
Extracellular water (%)	1.13–0.05	1.08	−1.72, 3.89	0.438	−0.046–0.28	−0.333	−3.17, 2.51	0.813	1.092–0.337	0.755	−2.08, 3.59	0.594

DID: Difference of differences. The *p* value is two tailed with statistically significance when < 0.05. * Significant difference of differences for men and women.
